# Ultrasound Combined With Microbubbles Loading BDNF Retrovirus to Open Blood–Brain Barrier for Treatment of Alzheimer’s Disease

**DOI:** 10.3389/fphar.2021.615104

**Published:** 2021-03-05

**Authors:** Feng Wang, Xi-Xi Wei, Lian-Sheng Chang, Lei Dong, Yong-Ling Wang, Na-Na Li

**Affiliations:** ^1^ Henan Key Laboratory of Medical Tissue Regeneration, School of Basic Medical Sciences, Xinxiang Medical University, Xinxiang, China; ^2^ Henan Key Laboratory of Neurorestoratology (The First Affiliated Hospital of Xinxiang Medical University), Xinxiang, China; ^3^ Department of Physiology and Pathophysiology, School of Basic Medical Sciences, Xinxiang Medical University, Xinxiang, China; ^4^ Department of Histology and Embryology, School of Basic Medical Sciences, Xinxiang Medical University, Xinxiang, China

**Keywords:** cationic microbubbles, Alzheimer’s disease, retrovirus, BDNF, blood-brain barrier

## Abstract

**Background:** Brain-derived nerve growth factor (BDNF) is a promising effective target for the treatment of Alzheimer’s disease (AD). BDNF, which has a high molecular weight, has difficulty in crossing the blood–brain barrier (BBB). The study aimed to prepare microbubbles loading brain-derived nerve growth factor (BDNF) retrovirus (MpLXSN-BDNF), to verify the characteristics of the microbubbles, and to study the therapeutic effect of the microbubbles combined with ultrasound on the opening of the blood–brain barrier in an AD rat model.

**Methods:** 32 adult male SD rats were randomly divided into four groups: control group, ultrasound + pLXSN-EGFP microbubble group (U + MpLXSN-BDNF), ultrasound + pLXSN-BDNF microbubble group, and ultrasound + microbubble + pLXSN-BDNF virus group (U + MpLXSN-BDNF), with eight rats in each group. At the same time, the left hippocampus of rats was irradiated with low-frequency focused ultrasound guided by MRI to open the blood–brain barrier (BBB). The effects of BDNF overexpression on AD rats were evaluated behaviorally before and 1 month after the treatment. The number of acetylcholinesterase (ChAT)-positive cells and the content of acetylcholine (ACh) in brain tissues were determined by immunohistochemistry and high-performance liquid chromatography (HPLC), respectively. IF staining of synaptic spines and Western blot of synaptophysin presented herein detected synaptic density recovery.

**Results:** Signal intensity enhancement at the BBB disruption sites could be observed on the MR images. The behavioral evaluation showed that the times of crossing the original platform in the U + MpLXSN-BDNF group increased significantly after treatment. Immunohistochemistry and HPLC revealed that the number of ChAT-positive neurons and the contents of ACh in the brain were significantly decreased in the treated groups compared with the controls. IF staining of synaptic spines and Western blot data of synaptophysin showed that the U + MpLXSN-BDNF group can recover the synaptic loss better by BDNF supplementation than the other treatment groups.

**Conclusion:** Ultrasound combined with viral microbubbles carrying BDNF can increase the transfection efficiency of brain neurons, promote the high expression of exogenous gene BDNF, and play a therapeutic role in the AD model rats.

## Introduction

Alzheimer’s disease (AD), an age-related neurodegenerative disease, is the main cause of dementia in the elderly (Marsh et al., 2016; Du et al., 2020). Current treatment of AD can only relieve symptoms, but cannot prevent or slow down the process of neurodegeneration (Wyss-Coray and Rogers, 2012; Guillot-Sestier and Town, 2013). Therefore, new treatment techniques and methods are needed. Brain-derived nerve growth factor (BDNF) can affect learning memory by regulating the synaptic plasticity of the hippocampus and the cholinergic nervous system in the prosencephalon (Leal et al., 2017; Kowianski et al., 2018; Bawari et al., 2019). Further study is required on the mechanism underlying the effect of BDNF on learning memory and to find effective targets for the treatment of AD.

BDNF, which has a high molecular weight, has difficulty in crossing the blood–brain barrier (BBB) (Han et al., 2000; Pardridge, 2007). The brain tissue can also be injured by direct injection of BDNF or by a viral vector carrying the BDNF gene. Therefore, finding a way to promote the noninvasive method of BDNF through BBB, as well as to increase its effective concentration in the central nervous system, has become an urgent problem for treatment of AD.

Ultrasound with microbubbles has been demonstrated to open the BBB locally, reversibly, and noninvasively at energy levels that do not cause cellular damage, which provides the possibility of successfully treating brain diseases, such as PD. Some scientists have used focused ultrasound with a microbubble contrast agent to open the BBB and treat central nervous system diseases by targeted release of drugs and biomolecules (Kobus et al., 2016; Alli et al., 2018; Zhao et al., 2018). Acoustic microbubbles could be used as gene carriers to treat diseases. Ultrasound-targeted microbubble destruction (UTMD) technology uses microbubble contrast agents as carriers to make an adherent surface or to encapsulate target genes (Tan et al., 2016; Chang et al., 2017). UTMD is definitely a promising strategy to improve the efficiency of gene delivery for multiple applications, and it is proven by increasing evidence that organs can be targeted with its high specificity (Danialou et al., 2002). Targeted acoustic microbubbles containing target genes are injected intravenously to reach target tissues, where the bubbles are broken to release the genes using ultrasonic irradiation.

Targeted delivery by opening BBB locally, reversibly, and noninvasively by using focused ultrasound combined with microbubbles carrying drugs or genes targeted provides a new strategy for the treatment of central nervous system and intracranial diseases (Sierra et al., 2017). In this study, retroviral ultrasound microbubbles loading BDNF (MpLXSN-BDNF) were prepared, and the therapeutic effect of combined treatment with ultrasound in the opening of BBB was studied, aiming to provide a new method for the treatment of AD.

## Materials and Methods

### Main Materials and Instruments

Experimental agents and instruments included 1,2-Distearoyl-sn-glycero-3-phosphocholine (DSPC, Avanti Polar Lipids Inc., Alabaster, AL, United States), 1,2-distearoyl-sn-glycero-3-phosphoethanolamine-N-[amino (polyethylene glycol)-2000] (DSPE-PEG2000, Avanti), polyethylenediamine-600 (PEI6000) (Avanti Polar Lipids Inc., Alabaster, AL, United States), SD rats (Medical Experimental Animal Center of Guangdong Province), automatic dilution AccuSizer particle counter (Particle Sizing Systems, Santa Barbara, CA, United States), inverted fluorescence microscope (Olympus, Japan), centrifuge (Eppendorf, United States), and rabbit anti-ChAT (Choline acetyltransferase) antibody (Thermo Fisher Scientific, Rockford, Illinois, United States). The system used to generate ultrasound energy in all the experiments comprised a function generator (AGF3022B; Tektronix, United States), an RF amplifier (DC2500A; AR, Souderton PA, United States), and a custom-made passive L–C matching circuit. Ultrasound waves were generated using a single-element focused ultrasound transducer (Valpey Fisher, Hopkinton, MA, United States). The subsequent experiments were performed under these conditions as our previous research (frequency, 1 MHz; MB dosage, 0.5 ml; exposure time, 1 min; pressure amplitude, 0.8 MPa; delay time, 60 s) (Wang et al., 2012).

### Preparation of Cationic Microbubbles

DSPC, DSPE-PEG2000, and PEI600 were placed proportionally (molar ratio 9:0.5:0.5) in a test tube. A thin layer of phospholipid was formed on the tube wall under the action of nitrogen flow (0.1 Mpa). The orifice of the test tube was sealed with a film, and holes were made with a needle on the top of the film. The tube was put in a 500-ml suction flask and vacuumed for 2–3 h. 5 ml Tris buffer was added to the test tube. The phosphatide suspension was separated into 2.5 ml vial with 1 ml per bottle after ultrasonic oscillation at 55–60°C for 20 min, and the gas exchange was carried out. The vial bottles were filled with perfluoropropane (Chang et al., 2017). Ordinary lipid microbubbles and biotinylated lipid microbubbles were prepared by the above methods.

### Preparation of Cationic Microbubbles Carrying Brain-Derived Nerve Growth Factor Retrovirus (MpLXSN-BDNF)

The microbubbles were prepared by mechanical oscillation and washed by the centrifugal floating method with PBS solution 3–4 times to remove the phospholipid that did not form microbubbles (Jin et al., 2013). 4 × 10^7^ microbubbles were added to 1 ml virus (4.2 × 10^9^ particles/mL) and incubated at room temperature for 30 min. Then, microbubbles were washed twice by a floating method to remove the unbound virus particles, and the retroviral vector pLXSN-BDNF microbubble contrast agent was obtained.

Four sample aliquots were taken from the virus solution, washing the supernatant each time and microbubble solution binding virus, in which the virus quantity was detected by real-time (fluorescence) quantitative PCR, and the adhesion efficiency of the virus and microbubbles was determined. The preparation and detection methods for pLXSN-EGFP microbubbles were the same as the above.

### Animal Grouping, Preparation, and Treatment of Alzheimer’s Disease Model

Thirty-two adult male SD rats (supplied by Medical Experimental Animal Center of Guangdong Province) weighing 200–250 g were randomly divided into the following groups: control group, ultrasound + pLXSN-EGFP microbubble group (U + MpLXSN-EGFP), ultrasound + pLXSN-BDNF microbubble group (U + MpLXSN-BDNF), and ultrasound + microbubble + pLXSN-BDNF virus group (U + M + pLXSN-BDNF), with eight rats in each group. All animal experimental protocols were reviewed and approved by the Institutional Animal Care and Use Committee of Xinxiang Medical University (Permit No. 19–108). The rats in each group were weighed before operation. After intraperitoneal injection with 10% chloral hydrate, the rats were fixed on a stereotaxic apparatus, and the skin was cut for about 2 cm to expose the anterior fontanelle. Location of the left ventricle (referring to the atlas of rat brain localization compiled by Paxinos and Watson): 2 mm behind the anterior fontanelle, 1.3 mm on the left, and 4 mm deep. A 10-µL microsyringe was connected with the micro-pump (Stoelting, UNITED STATES) to perform the injection into the left ventricle. The left ventricles of rats in the control group were injected with 5 μL normal saline, while rats in the other groups were injected with 5 μL Aβ (1–40) at 0.5 μL/min for 10 min. The needles were retained for 10 min after the injection. The skin was sutured and disinfected with iodophor and alcohol. Penicillin sodium was injected intraperitoneally at 4 × 10^4^ U/d for four consecutive days to prevent infection, and the AD rat models were replicated.

After successful preparation of the model, the rats in the U + MpLXSN-EGFP group were injected 4 × 10^7^ pLXSN-EGFP microbubbles through the tail vein. The rats in the U + MpLXSN-BDNF group were injected 4 × 10^7^ pLXSN-BDNF microbubbles through the tail vein. The rats in the U + M + pLXSN-BDNF group were injected 4 × 10^7^ microbubbles and pLXSN-BDNF virus through the tail vein. After the injection, the left hippocampus of rats was sonicated with low-frequency focused ultrasound to open BBB. The control group was not treated.

### MRI

The MRI scanner used was a standard 3.0 T Signa system (TRIO 3.0 T MRI; Siemens MAGNETOM, Erlangen, Germany). Anatomical images were acquired in multiple planes prior to and after sonication using a T2-weighted fast spin echo sequence (TE = 16 ms; TR = 1,000 ms; ETL = 4; BW = 16 kHz; matrix, 1846256; slice = 1 mm; NEX = 2; FOV = 5 cm) to evaluate whether signs of tissue damage were present after exposure. The rats were anesthetized with 30% chloral hydrate during the imaging procedure. A 7.5-cm diameter surface coil was placed under the head. Sonication was performed through a hole in the coil that was filled with a bag containing degassed water. A gradient echo sequence was used to aim the beam at the brain. Following each sonication, T2-weighted fast spin echo images were obtained and repeated after an intravenous bolus injection of meglumine gadopentetate MR contrast agent (0.1 ml/kg; Consun, Guangzhou, China) to detect and evaluate the opening of the BBB.

### Effects of Increased Expression of Brain-Derived Nerve Growth Factor in the Hippocampus on the Behavior of Alzheimer’s Disease Rat Model

After the AD model was established, the animals in each group were treated according to the method described in the preceding section. The Morris water maze test was performed before and 1 month after the treatment. The Morris water maze consists of a circular stainless steel pool (150 cm in diameter) filled with opaque white paint as previously described. The platform was set in the first quadrant, the water surface was 1.5 cm higher than the platform, and the water temperature was maintained at 19–20°C. The animals were then trained in the spatial learning task for four trials per day for six consecutive days. In each trial, the rats started from one of four quadrants facing the wall of the pool and ended when the animal climbed on the platform. If the mice did not locate the platform in 60 s, they were guided to the platform. The swimming path and the time used to find the platform (latency) were recorded by a video camera fixed to the ceiling of the room, 1.5 m from the water surface. Spatial memory was tested 2 d (day 8) after training. After removing the platform, the required time to reach the platform from the entry point within 60 s, the swimming distance, and the number of platform crossings were recorded (Dong et al., 2015; Du et al., 2019).

### Immunohistochemistry of ChAT-Positive Neurons in Each Group

The hippocampal tissues of rats in each group were routinely embedded with paraffin and dissected. The sections were immersed in 0.5% hydrogen peroxide solution at room temperature for 20 min, and were incubated with goat serum for 30 min to block specific antigens. Rabbit anti-ChAT antibody (diluted at 1:2000) was added and incubated overnight at 4°C. Samples were washed with PBS three times, and then sheep anti-rabbit IgG antibody was added and incubated in a dark box at room temperature for 1 h. Then, samples were washed with PBS three times, sealed, observed, and photographed under a microscope. Three sections and five high magnification view fields (400x) were taken from each section. ImagePro image analysis software was used to analyze the number of ChAT immunoreactive neurons. The neurons showing blue deposits in the cell bodies and dendrites were counted, and the average values were obtained.

### Immunofluorescence Staining of Synaptic Spines

To prepare the tissue for immunofluorescence (IF) staining, the rats were anesthetized and perfused transcardially by PBS and 4% paraformaldehyde. The brain of each mouse was removed and immersed in a 4% PFA solution for 2 h and dehydrated by gradients of 15, 20, and 30% sucrose. After preparing the cryosections, the tissues were incubated with the primary antibodies: SYP (1:500; Millipore, MAB5658). Alexa Fluor–conjugated secondary antibodies (1:300; Invitrogen) were used to recognize the primary antibodies. The sections were coverslipped with the mounting medium (Dako, CA, United States). All sections were examined by a laser scanning confocal microscope (Nikon C1–Si).

### Effect of Brain-Derived Nerve Growth Factor Expression on Hippocampal ACh Concentration in Rats by High-Performance Liquid Chromatography

The animals were decapitated, and about 0.5 g of the hippocampal tissue was frozen. After accurate weighing, the specimen was placed into the precooled tissue homogenizer with 5 ml of 0.5 mol/L perchloric acid added, homogenated, and centrifuged at 4°C, 20,000 g for 10 min. The supernatant was adjusted to pH 6 with 0.5 mol/L KOH solution. The supernatant was centrifuged once more and filtered with 0.45 μm pore size membrane. 5 μL of the liquid was taken, and the concentration of ACh was detected by high-performance liquid chromatography (HPLC).

### Statistical Analysis

SPSS 19.0 statistical software was used for data analysis. Measurement data were expressed as mean ± standard deviation $\left(\bar{\text{x}}\pm \text{SD}\right)$. Statistical analyses were performed by Student’s t test for two-group comparisons, one-way ANOVA, followed by *post hoc* tests for multiple comparisons among more than two groups. *p* < 0.05 was considered to be statistically significant.

## Results

### Detection of Ultrasound Microbubbles

**FIGURE 1:**
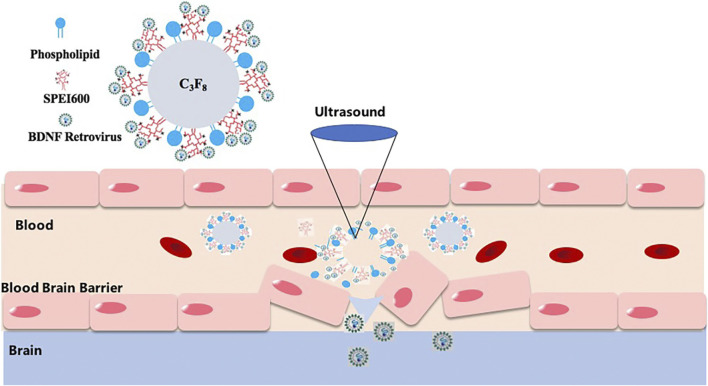
Technology road map of MRI-guided focused ultrasound-induced blood–brain barrier disruption to deliver BDNF retrovirus into the brain. BDNF retrovirus is released to the target cell in the brain after the microbubbles destroying and inducing blood–brain barrier disruption by MRI-guided focused ultrasound at the site of the target brain tissue.

**FIGURE 2:**
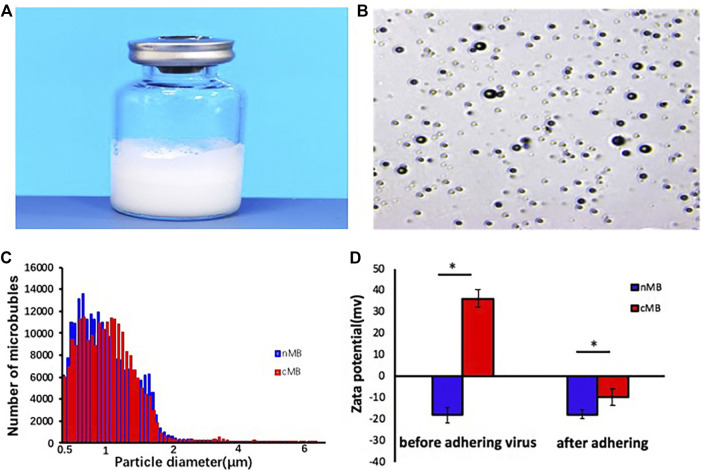
Characterization of virus-loaded microbubbles. **(A)** The microbubbles prepared in the vials. **(B)** Observation of microbubbles in a light field. **(C)** Particle size distribution of nMB and cMB. **(D)** Potential change in microbubbles after adding virus, nMB, and cMB.

A schematic illustration of the structure of M pLXSN-BDNF is presented in Figure 1, which showed BDNF retrovirus is released to the target cell in the brain after the microbubbles destroying and inducing blood–brain barrier disruption by MRI-guided focused ultrasound at the site of the target brain tissue. The average diameter of normal microbubbles (nMB) and the concentration of cationic microbubbles (cMB) were 1.05 ± 0.5 µm and 1.2 ± 0.3 × 10^10^ bubbles/mL, respectively (Figure 2C). Prepared microbubbles were observed under a microscope, and the average surface potential of the cationic microbubbles measured by Malvern laser particle counter was 36.3 ± 4.04 mv. As shown in Figure 2D, the potential changed to -9.67 ± 3.05 mv after the virus was added.

### Blood–Brain Barrier Disruption Induced by MRI-Guided Focused Ultrasound in Rats

**FIGURE 3:**
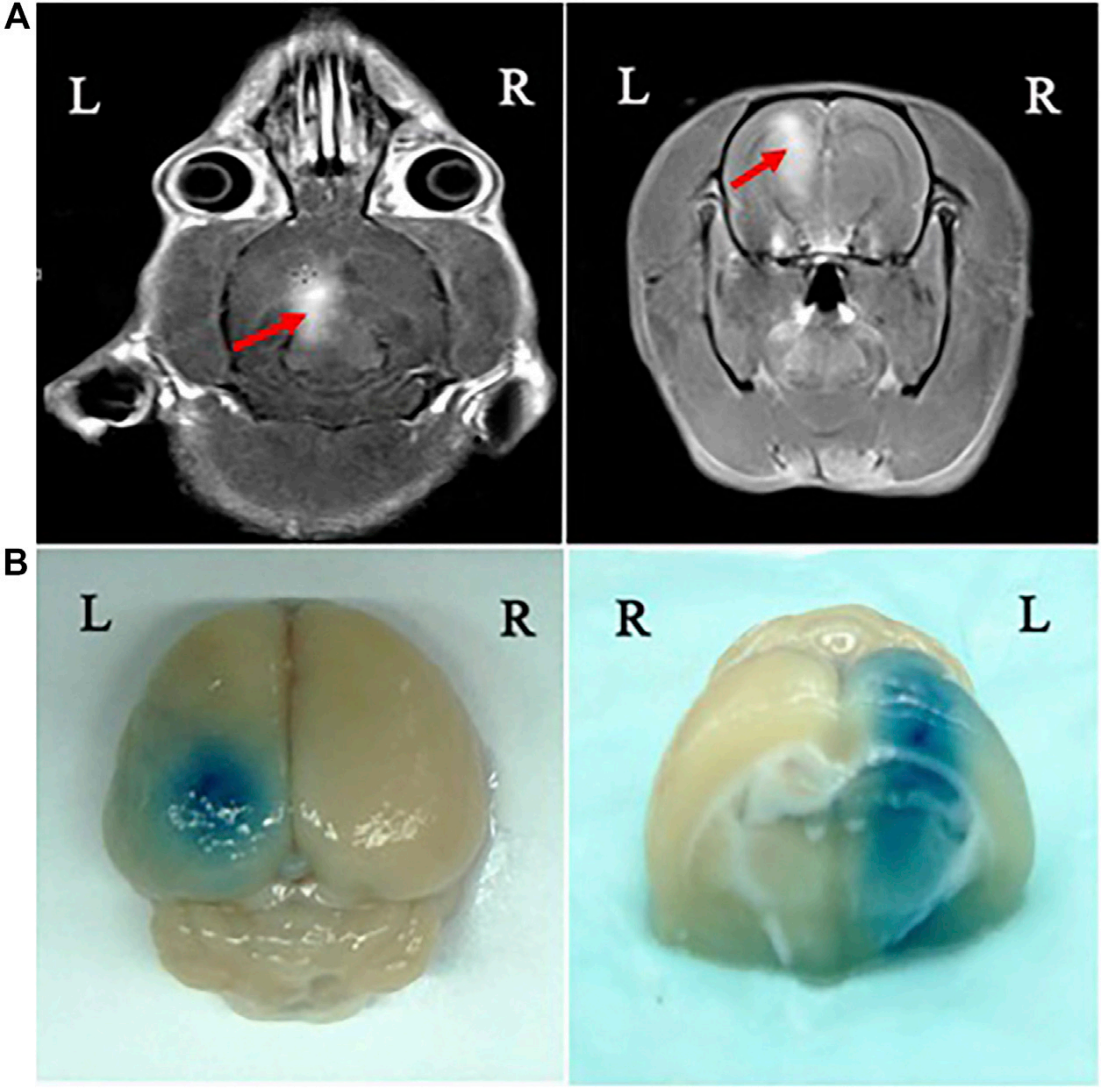
MRI monitoring of BBB disruption and photographs of the harvested brain showing BBB disruption induced by focused ultrasound. **(A)** BBB opening was monitored by leakage of the MR contrast agent into the brain parenchyma on axial (AX) and coronal (COR) MR images (red arrows). **(B)** The location of BBB opening was confirmed by EB staining of the affected area.

BBB disruption was observed in the focal zone of the ultrasound beam with EB extravasation. The opening of the BBB by MRI-guided focused ultrasound was evaluated under optimum parameters according to the findings described above. We monitored and confirmed BBB opening by MRI and leakage of the EB and MR contrast agent through the BBB into the cortex and caudate putamen of the brain after sonication. Leakage of the MR contrast agent to the brain parenchyma was observed on the MR images (Figure 3A). The brain of each animal was harvested 4 h after sonication. The location of the BBB opening was confirmed by EB staining of the affected area (Figure 3B).

### Behavioral Detection of Therapeutic Effect in Each Group

**FIGURE 4:**
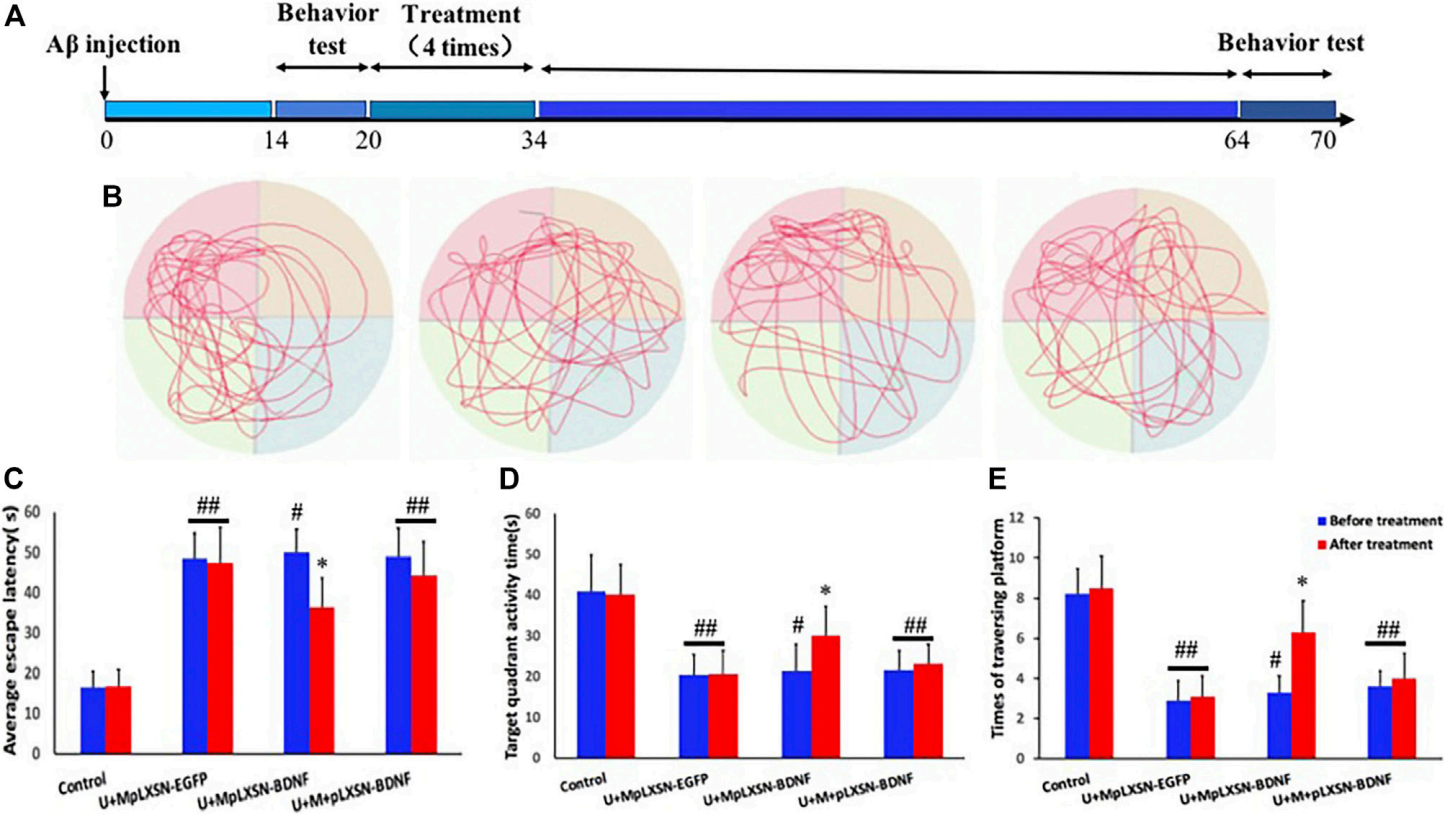
Morris water maze test results. **(A)** Schematics show the treatments. AD rat models were testing two weeks after injecting with Aβ (1–40). Then, every group was treated for 4 times (2 times per one week). A month after treatment, the cognitive behaviors were detected again. **(B)** The representative swimming tracks during the memory test carried out on day 8 by removing the hidden platform. **(C)** The average escape latency to find the platform tested on day 8. **(D)** Target quadrant activity time tested on day 8. **(E)** Target platform traversing times tested on day 8. *p < 0.05, compared with U + MpLXSN-BDNF group before treatment. #*p* < 0.05, compared with the control group. ##*p* < 0.01, compared with the control group.

Figure 4A is the schematic drawing of the time course in this study. The Morris water maze test was performed to evaluate the spatial learning and memory abilities of rats. We evaluated whether ultrasound combined with microbubbles carrying BDNF retrovirus ameliorate the spatial memory deficit in AD rats by using the Morris water maze (MWM) task at 4 weeks after treatments. Within six training sessions, the results indicated that the average escape latency to find the platform was significantly decreased after treatment in the U + MpLXSN-BDNF group, compared with other treatment groups (*p* < 0.05) (Figures 4B,C). Target quadrant activity time and target platform traversing times significantly increased after treatment in the U + MpLXSN-BDNF group, compared with other treatment groups (*p* < 0.05).

### Changes in Number of ChAT-Positive Neurons in Each Group Detected by Immunohistochemistry

**FIGURE 5:**
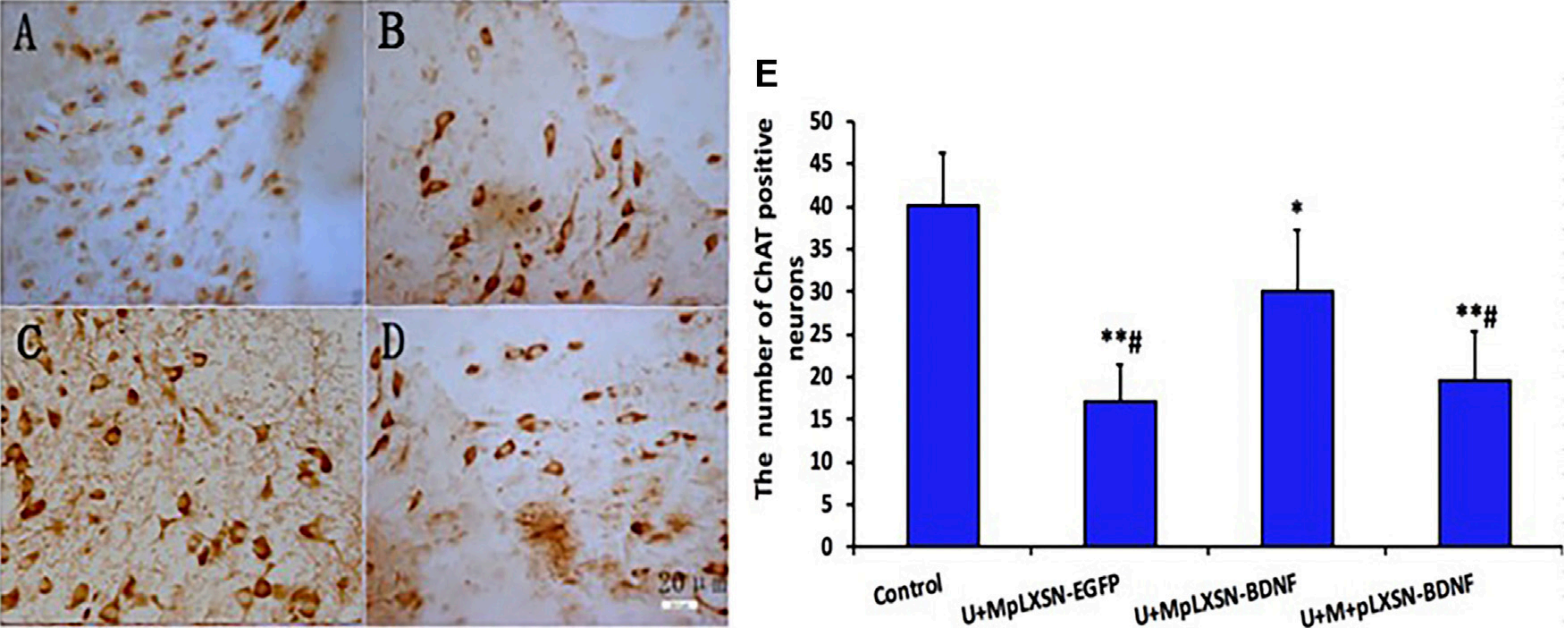
Microscopic effects of treatment on ChAT-positive neurons. **(A)** Normal. **(B)** U + M_pLXSN-EGFP_. **(C)** U + MpLXSN- BDNF. **(D)** U + M + pLXSN-BDNF (bar: 20 μm). **(E)** The numbers of ChAT-positive neurons in each group were analyzed by scanning software. *p < 0.05, **p < 0.01 compared with normal group, #*p* < 0.05 compared with the U + MpLXSN-BDNF group.

Results of immunohistochemistry showed that the numbers of ChAT-positive neurons in experimental groups were significantly decreased compared with that in the control group (*p* < 0.05 in U + M pLXSN-BDNF group, *p* < 0.01 in U + M pLXSN-EGFP group, and U + M + pLXSN-BDNF group), and were significantly decreased in the U + M pLXSN-BDNF and U + M pLXSN-EGFP groups (*p* < 0.05) as compared to the U + M pLXSN-BDNF group (*p* < 0.05) (Figure 5).

### Effects of Different Treatments on Synaptic Density in the Hippocampus

**FIGURE 6:**
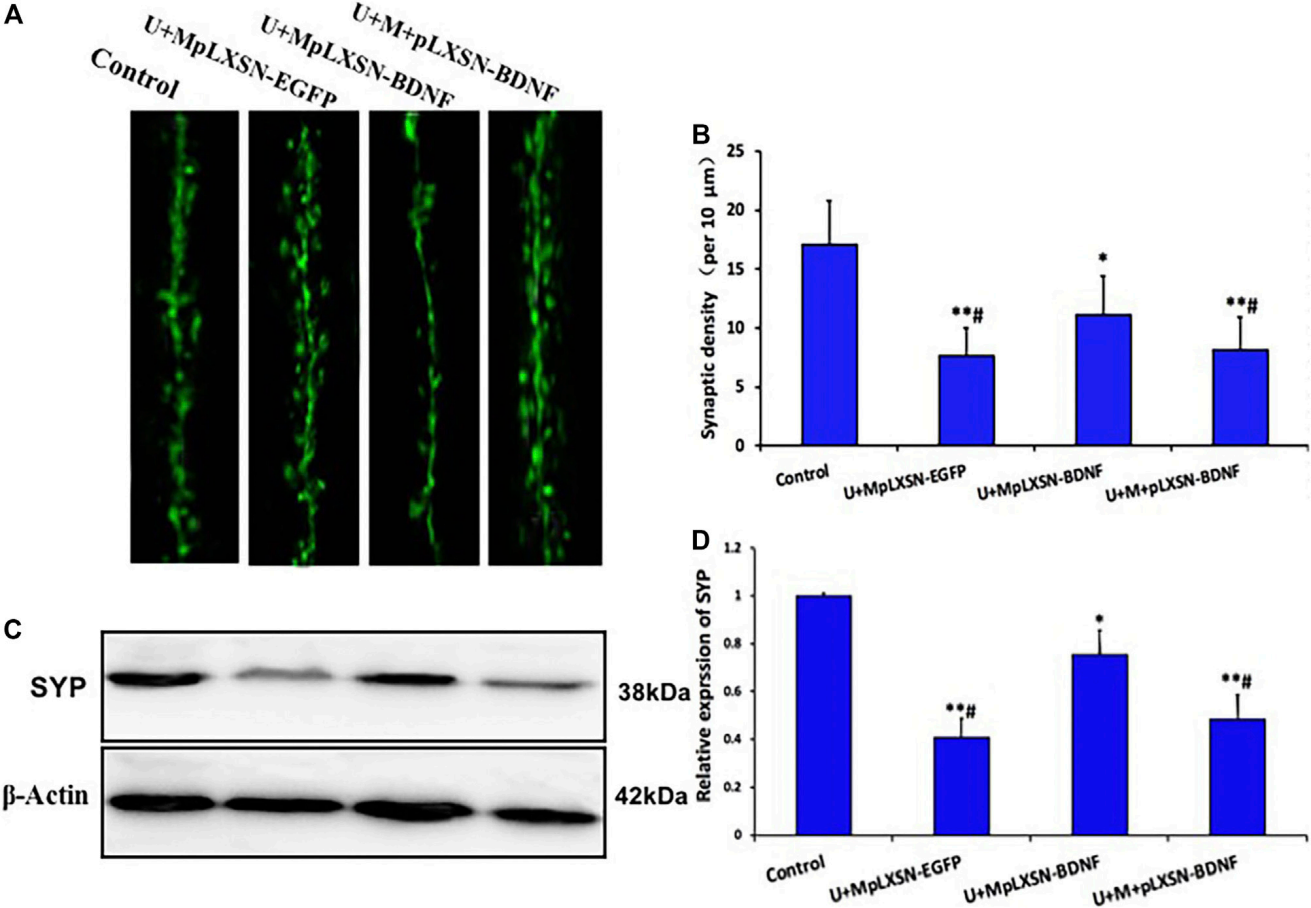
Overexpression of BDNF increases spine density in the hippocampus of the AD rat model. **(A)** Representative image of synaptic spines in each group (bar, 5 μm). **(B)** Quantification of the synaptic spine density (at least 15 neurons, three dendritic branches per neuron, from three mice were used for the analysis). **(C,D)** Representative Western blot data and quantification results for the hippocampal level of SYP in AD rats. *p < 0.05, **p < 0.01 compared with the normal group, ^#^ *p* < 0.05 compared with the U + MpLXSN-BDNF group.

To further characterize the role of different treatments on synaptic density in the hippocampus, we investigated the development of synaptic density in the hippocampus of every group by immunofluorescence staining. The data indicated that the U + M pLXSN-BDNF group displayed a higher density of synaptic spines, compared with U + M pLXSN-EGFP and U + M + pLXSN-BDNF groups (Figures 6A,B, *p* < 0.05). Furthermore, we determined the synaptic protein level of hippocampal lysates by Western blot data of synaptophysin (SYP). The data confirmed that the U + M pLXSN-BDNF group recovered more of the hippocampal level of synaptophysin than U + M pLXSN-EGFP and U + M + pLXSN-BDNF groups (Figures 6C, D, *p* < 0.05).

### Effects of Different Treatments on ACh Concentration in the Central Nervous System

**FIGURE 7:**
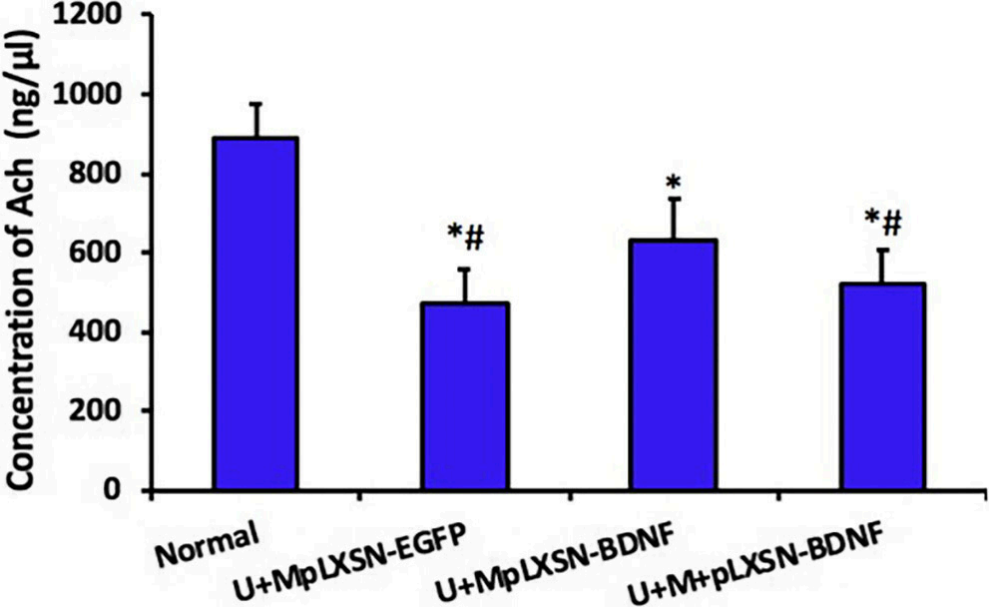
HPLC detection of Ach concentration in each group. *p < 0.05, **p < 0.01 compared with the normal group, ^#^ *p* < 0.05 compared with the U + MpLXSN-BDNF group.

HPLC revealed that the cerebral concentration of ACh in the experimental groups was significantly lower than that of the normal group (U + M pLXSN-EGFP and U + M + pLXSN-BDNF, *p* < 0.01; U + M pLXSN-BDNF, *p* < 0.05). The concentration of ACh in the U + M pLXSN-EGFP and U + M + pLXSN-BDNF groups was significantly reduced compared with the U + M pLXSN-BDNF group (*p* < 0.05) (Figure 7).

## Discussion

The neuropathological features of AD are argyrophilic senile plaques formed by abnormal deposition of β-amyloid protein (Aβ), neurofibrillary tangles, and the disappearance of a large number of synaptic connections in the hippocampus and temporal cortex. The role of neurotrophic factors, typically brain-derived neurotrophic factor (BDNF), in the treatment of AD has attracted recent attention with the development of cellular and molecular biology. BDNF can regulate the synaptic plasticity of the hippocampus and the cholinergic nervous system in the prosencephalon, affecting learning memory (Allen et al., 2013; Song et al., 2015; Jiao et al., 2016; De Pins et al., 2019). However, because of its high molecular weight (12.3 kDa), BDNF cannot cross the BBB, and direct intracerebral injections of BDNF or viral vectors carrying the BDNF gene may injure the brain tissue (Han et al., 2000). Therefore, it is imperative to find a method for the noninvasive delivery of BDNF across BBB and to increase its effective concentration in the central nervous system. Ultrasound combined with microbubbles can open BBB locally, reversibly, and noninvasively, which provides a new path for the clinical treatment of intracranial diseases.

The basic research on MRI-guided focused ultrasound combined with viral microbubbles in the opening of BBB and delivery of BDNF has not yet been reported in the treatment of AD. In this study, MRI-guided real-time focused ultrasound combined with retroviral microbubbles carrying the BDNF gene was used to irradiate the hippocampus of rats to open BBB, to promote retrovirus to cross the BBB, to transfect the nervous cells in this region, and to increase the expression of BDNF.

MRI has provided the necessary guidance for ultrasound-induced BBB disruption studies, including the placement of ultrasonic focus within the brain and assessment of BBB opening (Wang et al., 2012). In the present study, MRI was used for real-time monitoring of the site of BBB opening induced by ultrasound combined with MBs. Signal intensity enhancement at the BBB disruption sites could be observed on the MR images, suggesting that BBB disruption could be predicted by the obtained MR images (Wang et al., 2012).

The effect of increased intracranial BDNF on AD rats was evaluated by behavioral observation. One month after treatment, the Morris water maze showed that the target quadrant activity time and target platform traversing times in the U + MpLXSN-BDNF group increased significantly and the average escape latency to find the platform in the U + MpLXSN-BDNF group was significantly decreased after treatment.

Synaptic loss is another pathologic hallmark of the AD brain that is highly correlated with the pathogenesis of the disease (Gylys et al., 2004; Dong et al., 2015; Lee et al., 2015; Mcdole et al., 2015). Since BDNF regulates neurite outgrowth and synaptic plasticity, the reduction of the BDNF level is also involved in this pathophysiology. However, synaptic dysfunction and synapse loss, unlike neuronal loss, are reversible, highly dynamic, and plastic (Trachtenberg et al., 2002; Matsuzaki et al., 2004). IF staining of synaptic spines and Western blot data of synaptophysin presented herein reinforce this concept that synaptic density recovery is associated with the BDNF level and cognitive amelioration, providing evidence that the U + MpLXSN-BDNF group can recover the synaptic loss better by BDNF supplementation than the other treatment groups.

To further verify the effect of BDNF overexpression on ACh in the central nervous system and its protective effect on cholinergic neurons, the number of ChAT-positive neurons and the content of ACh in cerebral tissues were detected by immunohistochemistry and HPLC. Immunohistochemical results showed that the number of ChAT immunoreactive neurons in the U + M pLXSN-BDNF group was significantly higher than those in the U + M pLXSN-EGFP and U + M + pLXSN-BDNF groups. HPLC results also showed that, compared with U + M pLXSN-EGFP and U + M + pLXSN-BDNF groups, the content of cerebral ACh in the U + M pLXSN-BDNF group significantly increased, suggesting that U + MpLXSN-BDNF displayed significant therapeutic effect on the AD rat model. These results indicate that ultrasound, combined with viral microbubbles carrying BDNF, could increase the transfection efficiency of neurons in the brain and thus increase the expression of exogenous BDNF gene, which played a therapeutic role in the AD rat model.

In summary, we investigated the mechanism of BDNF in the treatment of AD. The long-term cerebral expression of target genes after noninvasive and reversibly opening of the BBB was studied by using low-frequency focused ultrasound combined with microbubbles and gene transfection technologies. The unique advantages of neurotrophic factors in the treatment of brain diseases were also elaborated, which has provided a new experimental basis for the clinical treatment of AD.

## Data Availability

The original contributions presented in the study are included in the article/Supplementary Material; further inquiries can be directed to the corresponding authors.
